# Thermal priming enhances heat tolerance in alfalfa (*Medicago sativa* L.) through activation of multiple metabolic pathways

**DOI:** 10.1186/s12870-025-07726-w

**Published:** 2025-12-09

**Authors:** Chengmin Jin, Jingliang Sun, Jinrui Zhao, Ziyao Zhang, Han Lu, Jixiang Zou, Hua Jin

**Affiliations:** College of Environment and Resource, Dalian Minzu University, No 18, Liaohe Road, Dalian, Liaoning 116600 China

**Keywords:** Thermal priming, Alfalfa, Multivariate analysis, Brassinosteroid, Spliceosome, Metabolic pathway

## Abstract

**Background:**

Elevated environmental temperatures disrupt plant physiological homeostasis, imposing thermal stress that severely compromises growth and development. While thermal priming - a brief exposure to sublethal high temperature has been shown to enhance subsequent heat stress tolerance in plants, the underlying molecular mechanisms remain poorly characterized. In this study, we employed an integrated physiological, transcriptomic and metabolomic approach to investigate how thermal priming [37℃ for 2 h (P1) followed by 43℃ for 2 h (P2), designated P3] improves heat tolerance in alfalfa (*Medicago sativa* L.) compared to unprimed controls (UP) exposed directly to 43℃.

**Results:**

Physiological analyses revealed that thermal priming significantly enhanced lodging resistance while increasing superoxide dismutase (SOD) and catalase (CAT) activities and reducing malondialdehyde (MDA) accumulation, indicative of improved oxidative stress management. Transcriptome profiling identified 1,267 upregulated genes in primed plants (P3 vs UP), with 47.9% being activated during the initial priming phase. Cluster analysis demonstrated stage-specific pathway activation: brassinosteroid (BR) signaling, spliceosome activity, glutathione metabolism and fatty acid metabolism pathways were rapidly induced during early priming (P1), while phenylpropanoid biosynthesis was activated later during the second phase (P2). Metabolomic analyses provided further mechanistic insights, showing that thermal priming triggered significant lignin accumulation in stems, enhanced activity of the ascorbate-glutathione (AsA-GSH) cycle with increased antioxidant levels, and elevated content of unsaturated fatty acids including erucic acid, linolenic acid and oleic acid, suggesting membrane lipid remodeling.

**Conclusions:**

Our findings demonstrate that thermal priming establishes a multi-faceted defense system in alfalfa through BR-mediated signaling. This coordinated response involves activation of the AsA-GSH cycle for reactive oxygen species (ROS) scavenging, upregulation of phenylpropanoid biosynthesis for structural reinforcement through lignin deposition, accumulation of unsaturated fatty acids to maintain membrane stability, and enhancement of spliceosome activity to ensure proper processing of heat-responsive transcripts. The sequential activation of these pathways during the priming phases creates a ‘stress memory’ that prepares plants for subsequent heat challenges. These insights advance our understanding of thermal priming mechanisms and provide potential targets for improving crop heat tolerance through molecular breeding strategies.

**Supplementary Information:**

The online version contains supplementary material available at 10.1186/s12870-025-07726-w.

## Background

Thermal priming, the process by which brief exposure to non-lethal high temperatures enhances subsequent heat stress tolerance, represents a critical adaptive strategy in plants [1]. This phenomenon has been demonstrated to improve thermotolerance through coordinated physiological, biochemical, and molecular responses [2]. Evidence from multiple species confirms the protective effects of thermal priming: in rice, priming at 28℃ maintained membrane integrity and biomass accumulation while reducing ROS and MDA levels during heat stress [3]. Similarly, brassica plants primed at 40℃ exhibited increased soluble sugars, CAT and peroxidase (POD) activities, along with reduced membrane damage when exposed to 45℃ stress [4]. Other studies have documented enhanced photosynthetic efficiency in azalea [5], improved antioxidant capacity in cape gooseberry [6], and preserved photosynthetic function in grapes following thermal priming [7].

At the molecular level, thermal priming induces sustained expression of small heat shock proteins (sHSPs) such as HSP21, HSP22, and HSP17.6 C, with primed plants showing more rapid and robust induction of these molecular chaperones [8]. In *Arabidopsis*, priming establishes epigenetic memory through JMJ-mediated demethylation of H3K27me3 at HSP loci, enabling faster transcriptional responses to subsequent heat stress [8]. These findings collectively demonstrate that thermal priming enhances thermotolerance through multiple mechanisms including photosynthetic protection, osmotic adjustment, and antioxidant defense.

Alfalfa (*Medicago sativa* L.), the “king of forages,” represents a globally important perennial legume valued for its nutritional quality, palatability, and environmental benefits in soil improvement and ecological restoration [9]. While adapted to warm climates (optimal growth at 18–22℃), rising global temperatures now pose significant challenges to alfalfa production [10]. Heat stress impairs alfalfa at multiple developmental stages, reducing seed germination rates, compromising photosynthetic efficiency, disrupting water relations, and inducing oxidative damage through ROS accumulation [11]. These physiological disturbances lead to decreased accumulation of critical nutrients including dry matter, carbohydrates, and starch [12], with yield reductions estimated at 17% per 1℃ temperature increase [13].

Despite extensive documentation of thermal priming effects across plant species, the specific mechanisms underlying this phenomenon in alfalfa remain poorly understood. This study employed the heat-tolerant cultivar ‘Sanditi’ and aimed to characterize morphological and physiological responses to thermal priming under heat stress; to identify differentially expressed genes and metabolic pathways through transcriptomic and metabolomic analyses; and to elucidate the molecular basis of priming-enhanced thermotolerance. Our integrated approach provides novel insights into alfalfa’s adaptive strategies against recurrent high-temperature stress and establishes a framework for improving heat tolerance in this critical forage crop.

## Results

### Screening of heat-resistant alfalfa varieties

**Fig. 1:**
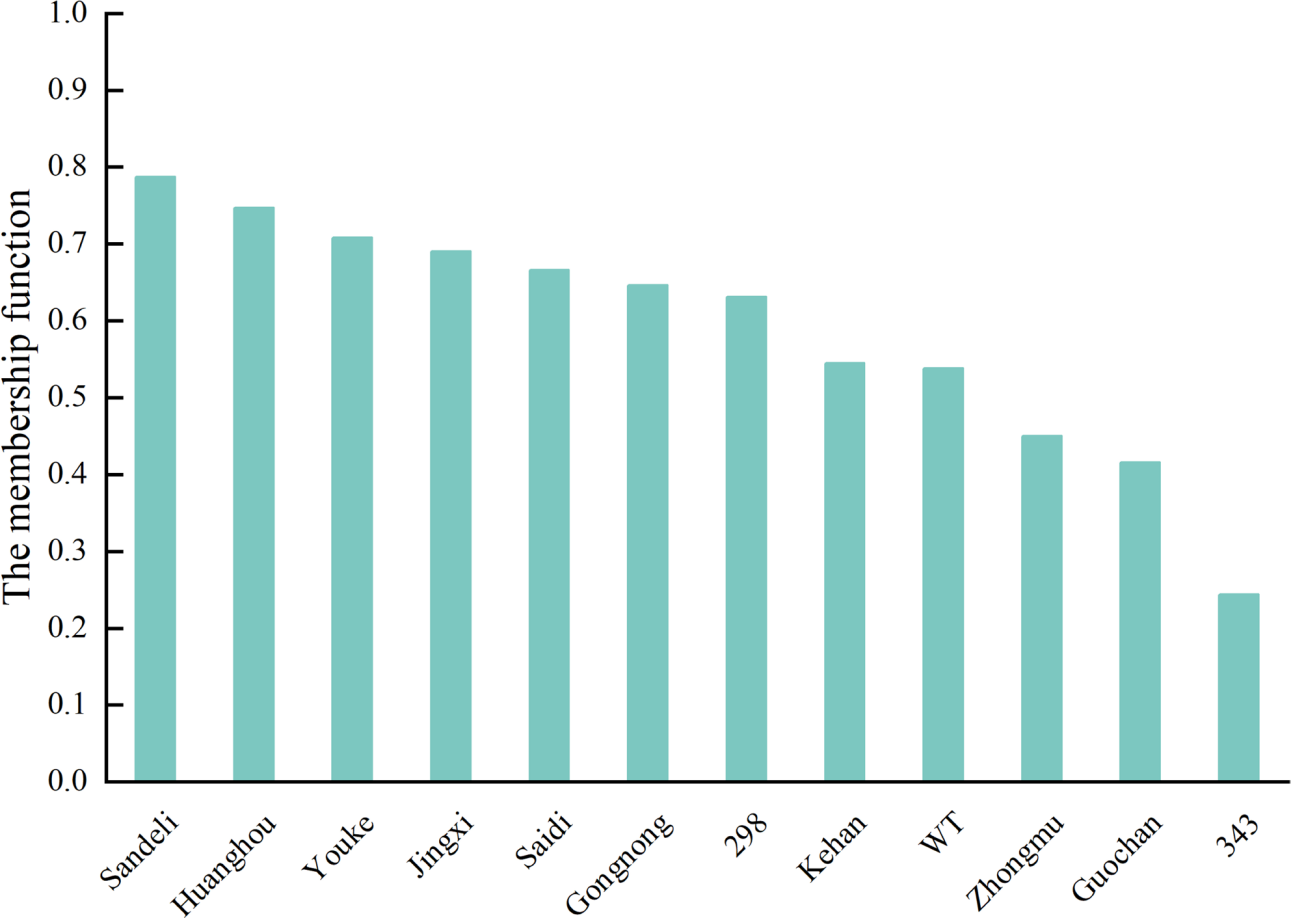
Comprehensive evaluation of heat tolerance in alfalfa varieties under heat stress conditions. The membership functions of 12 alfalfa indexes were accumulated and the average value was obtained. In order to avoid errors caused by variety differences, Xj, Xmax and Xmin are all calculated by “relative values” instead of “measured values”. Relative value = stress group/control group

To identify suitable germplasm for thermal priming studies, we evaluated the heat tolerance of 12 alfalfa varieties using a comprehensive membership function approach. This analysis incorporated multiple physiological indicators including chlorophyll content, MDA accumulation, electrolyte leakage (EL), and key antioxidant enzyme activities (POD, SOD, CAT) under 37℃ heat stress (Fig. 1, Table S1).

The screening revealed significant variability in thermotolerance among cultivars, with membership scores ranging from 0.244 to 0.763. ‘Sanditi’ emerged as the most heat-resistant variety (score: 0.763), demonstrating superior maintenance of chlorophyll content, reduced oxidative damage (lower MDA and EL), and enhanced antioxidant enzyme activities compared to other cultivars. In contrast, cultivar ‘343’ showed the poorest performance (score: 0.244). Based on these results, we selected ‘Sanditi’ for all subsequent thermal priming experiments due to its robust heat stress responses.

### Effect of thermal priming on alfalfa growth morphology

**Fig. 2:**
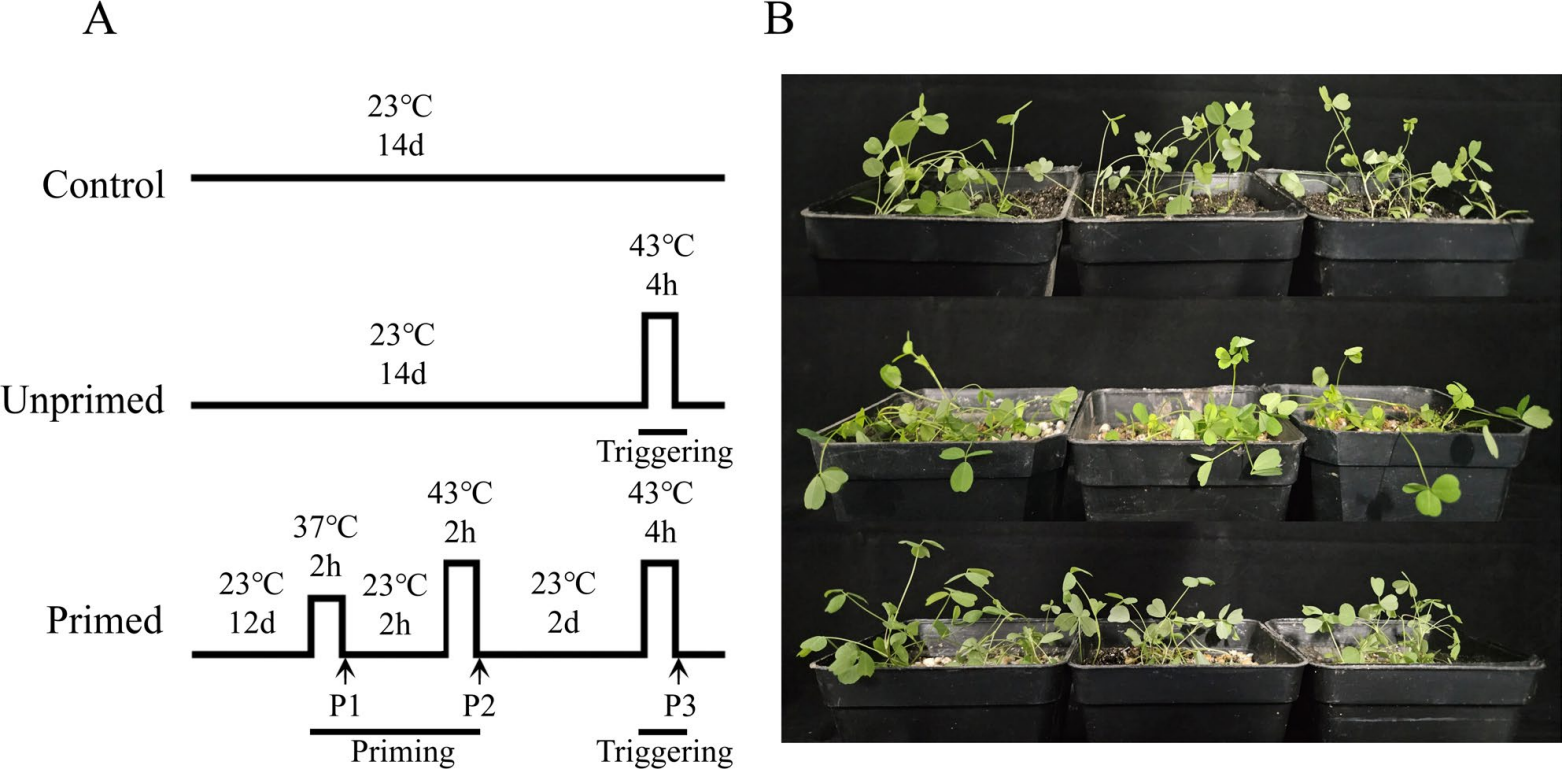
Treatment methodology and phenotypic response. **A** Experimental protocols. **B** Effect of thermal priming on alfalfa growth. Control: seedlings prior triggering; unprimed: Seedlings are triggered by heat stress; primed: Seedlings are subjected to heat priming and heat stress triggering

To investigate the impact of thermal priming on alfalfa growth phenotypes, seedlings were subjected to the heat priming (Fig. 2A). The results showed that both non-primed (UP) and primed (P3) groups exhibited wilting and lodging following heat stress, whereas control (CK) plants maintained normal morphology. Notably, P3 group demonstrated significantly reduced lodging severity (*P* < 0.05) and less pronounced leaf wilting compared to UP group (Fig. 2B). These findings suggest that thermal priming can effectively mitigate heat stress-induced morphological impairments in alfalfa.

**Fig. 3:**
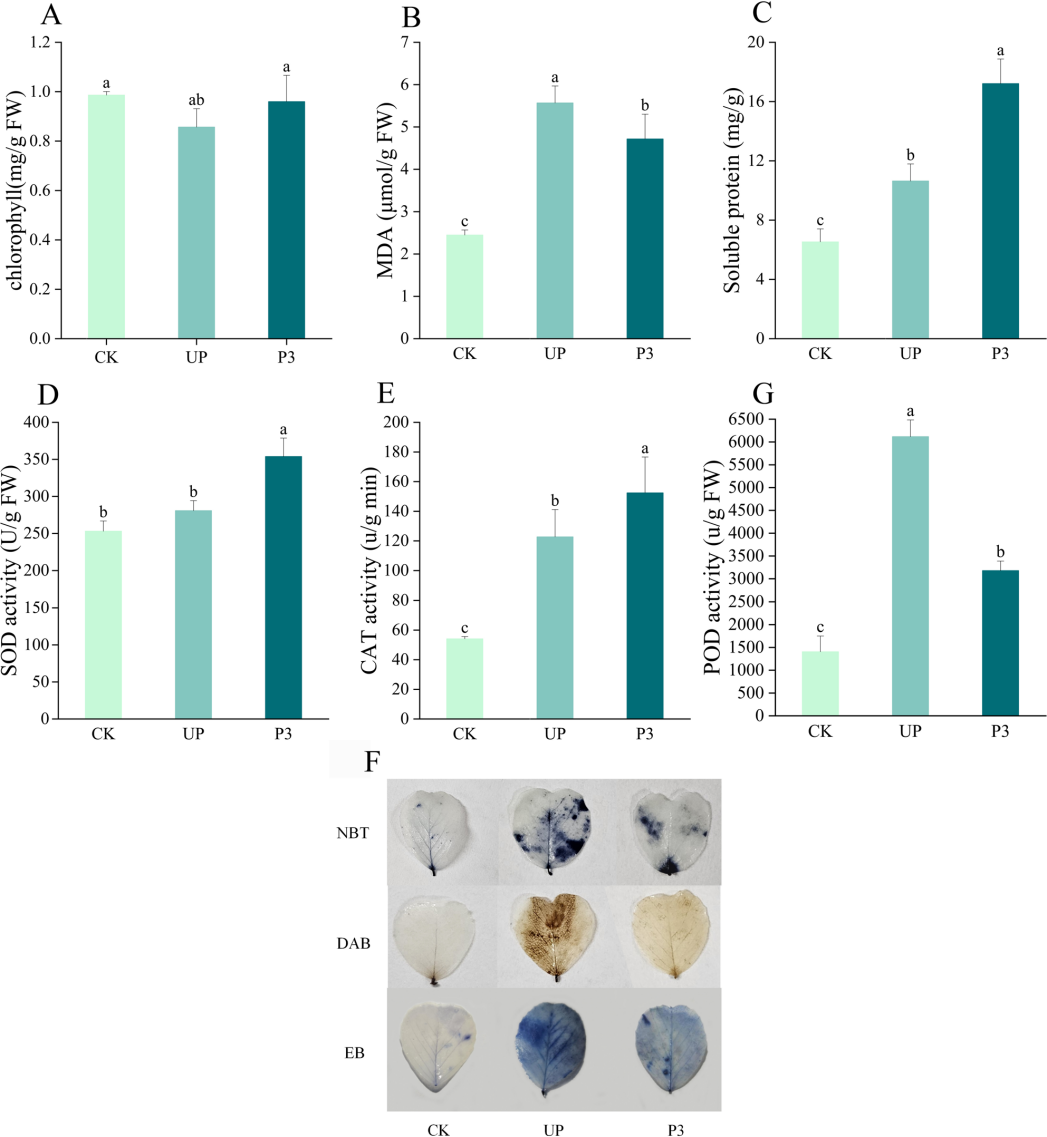
Effect of thermal priming on physiological index of alfalfa leaves under heat stress conditions. **A** Chlorophyll content. **B** MDA content. **C** Soluble protein content. **D** SOD activity. **E** CAT activity. **F** POD activity. **G** ROS generation as shown by histochemical staining. Data were expressed as the mean ± standard error of three independent biological replicates. Different letters indicated significant differences of *P* < 0.05 according to Duncan test

To assess the physiological impact of thermal priming on alfalfa, we measured chlorophyll content, MDA levels, soluble protein content, and antioxidant enzyme activities (SOD, POD, CAT) in seedlings from the CK (control), UP (unprimed + heat stress), and P3 (primed + heat stress) groups. Chlorophyll content and soluble protein levels were significantly higher in P3 than UP (Figs. 3A, C), suggesting that priming helps maintain photosynthetic efficiency and protein stability under stress. MDA content, a marker of lipid peroxidation, significantly increased in both UP and P3 groups compared to CK, confirming heat-induced membrane damage. However, P3 exhibited 18% lower MDA levels than UP (*P* < 0.05), indicating that thermal priming mitigates heat stress-induced membrane damage (Fig. 3B). SOD, POD, and CAT activities were also elevated in UP and P3 compared to CK. P3 showed a more pronounced increase in SOD and CAT but reduced POD activity when compared to UP (Figs. 3D-F), indicating that thermal priming selectively enhances key ROS-scavenging enzymes, reducing oxidative damage. To evaluate ROS generation and cellular integrity under heat stress, we performed histochemical staining. Nitrotetrazolium blue chloride (NBT), diaminobenzidine (DAB) and Evans blue (EB) staining revealed lighter staining in P3 vs UP, demonstrating that thermal priming reduces ROS accumulation and cell damage (Fig. 3G).

### Transcriptome profiling of thermal priming effects

**Fig. 4:**
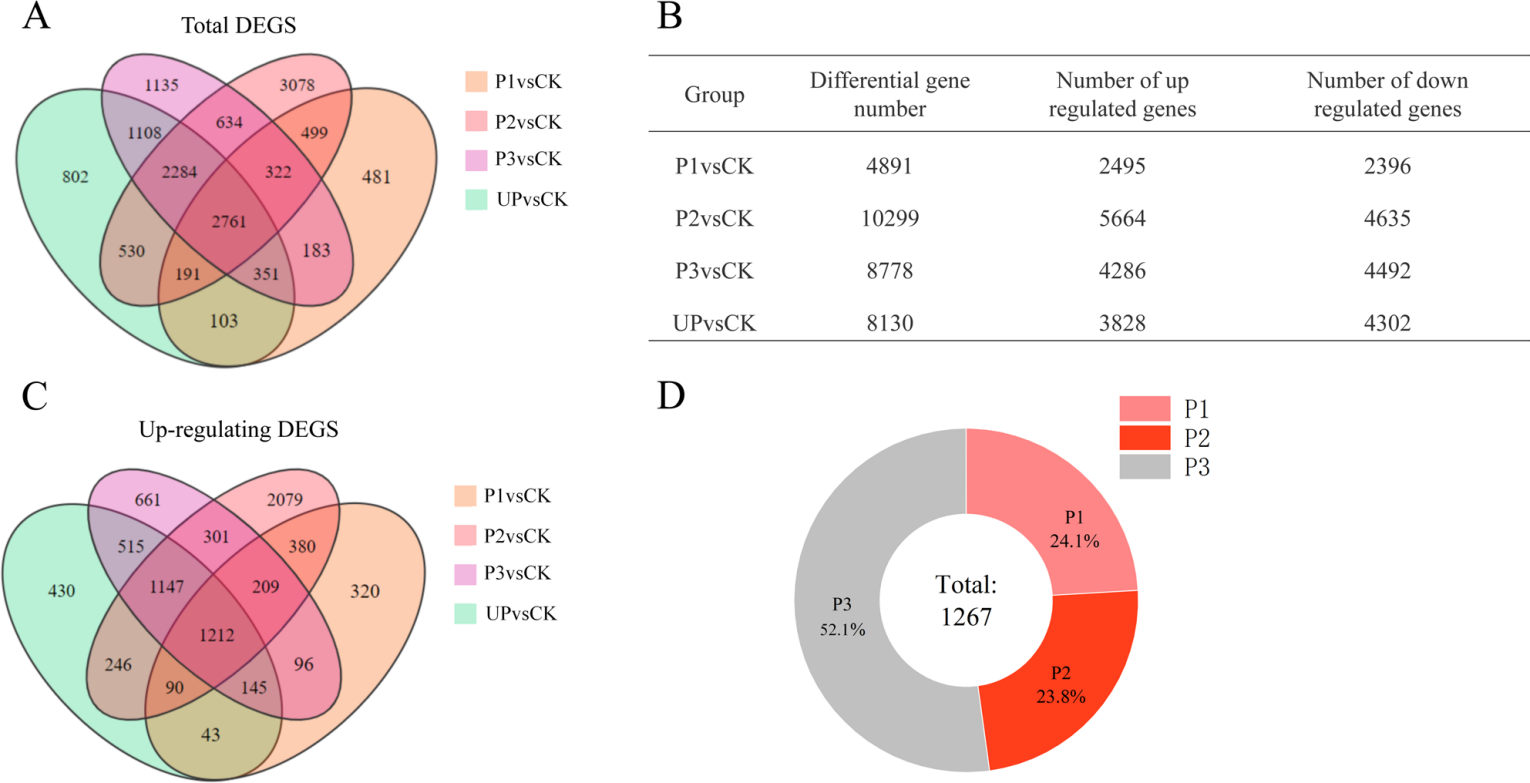
Number of differentially expressed genes in different treatment groups. **A** Venn diagram of total DEGs in each treatment group. **B** Number of DEGs in each treatment group. **C** Venn diagram of upregulated DEGs in each treatment group. **D** Expression patterns of upregulated DEGs in the P3 vs UP group

To investigate the molecular mechanisms underlying thermal exercise-induced heat tolerance in alfalfa, we performed transcriptome sequencing on leaves from five experimental groups: CK, UP, P1, P2, and P3. The sequencing generated a total of 101.15 GB of high-quality clean data, with each sample producing at least 6 GB of data. All samples exhibited Q30 scores exceeding 94% and GC content ranging from 42.11% to 42.95%, confirming the high quality of the transcriptome sequencing data (Table S2). Differential gene expression analysis (|FoldChange| ≥ 1 and FDR < 0.05) identified: P1 vs CK: 4,891 differentially expressed genes (DEGs; 2,495 up-regulated and 2,396 down-regulated); P2 vs CK: 10,299 DEGs (5,664 up-regulated and 4,635 down-regulated); P3 vs CK: 8,778 DEGs (4,286 up-regulated and 4,492 down-regulated); UP vs CK: 8,130 DEGs (3,828 up-regulated and 4,302 down-regulated) (Figs. 4A-C). These results clearly demonstrate that thermal exercise significantly alters gene expression profiles in alfalfa under heat stress conditions.

### Gene expression patterns during thermal priming

To elucidate the specific effects of thermal priming on alfalfa heat tolerance, we analyzed 1,267 differentially expressed genes (DEGs) that were uniquely up-regulated in the P3 group. These DEGs were categorized based on their temporal expression patterns during the thermal exercise regimen. Totally 305 DEGs (24.1%) showed initial upregulation during P1 phase, 301 DEGs (23.8%) exhibited subsequent upregulation during P2 phase, and 661 DEGs (52.1%) demonstrated late-stage upregulation during P3 phase (Figs. 4C-D). Notably, 47.9% of the total up-regulated DEGs (606 genes) were activated during the thermal priming period (prior to P3), while the remaining 52.1% showed delayed response. This biphasic pattern suggests both immediate and progressive transcriptional responses to thermal conditioning.

### Expression patterns of DEGs in alfalfa after thermal priming treatment

**Fig. 5:**
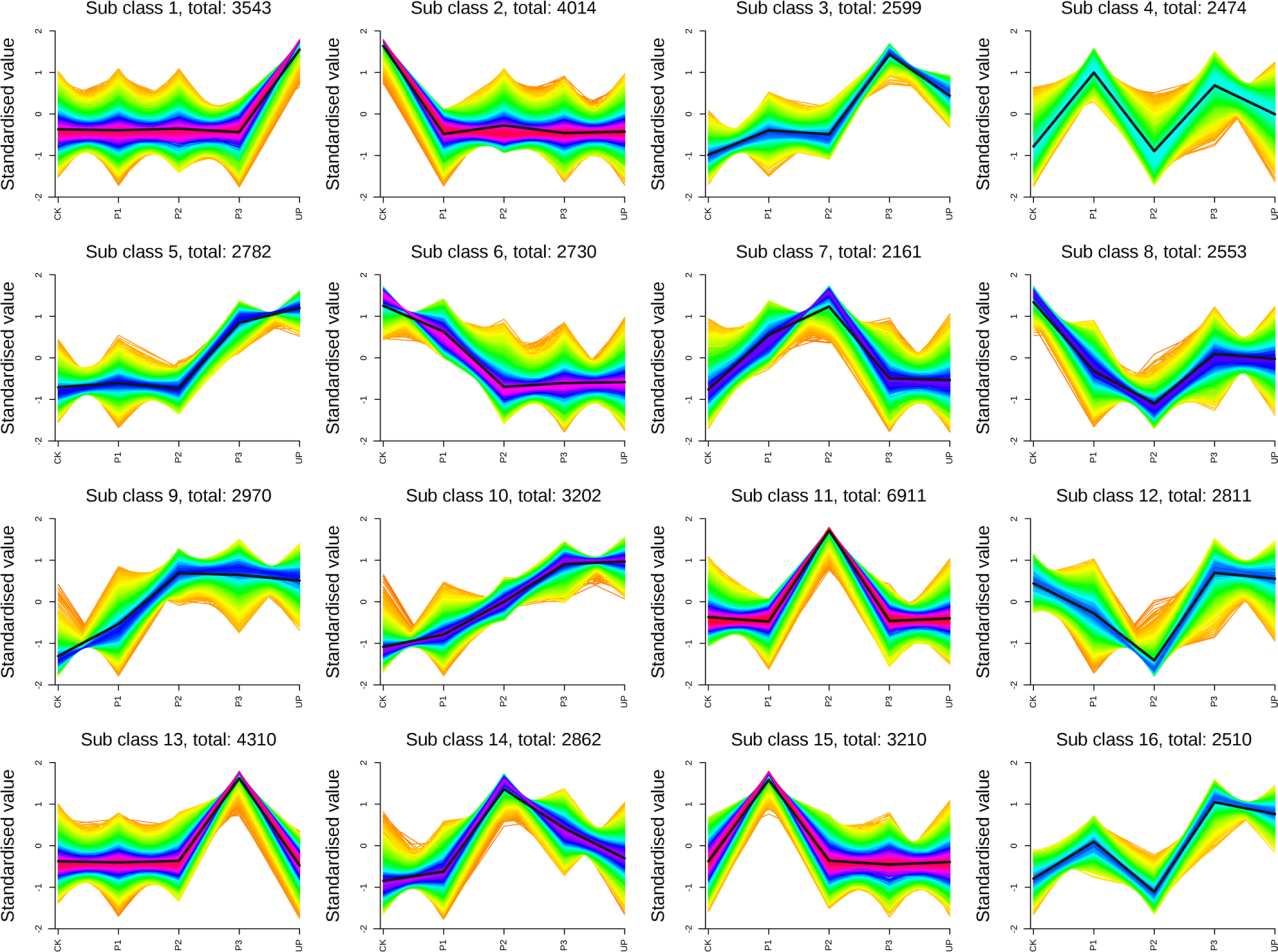
The expression patterns of DEGs under different treatments in leaves. Sub class represents gene groups with the same changing trend, and the number after total represents the number of genes in each group. The cluster centers are marked as the black lines

**Fig. 6:**
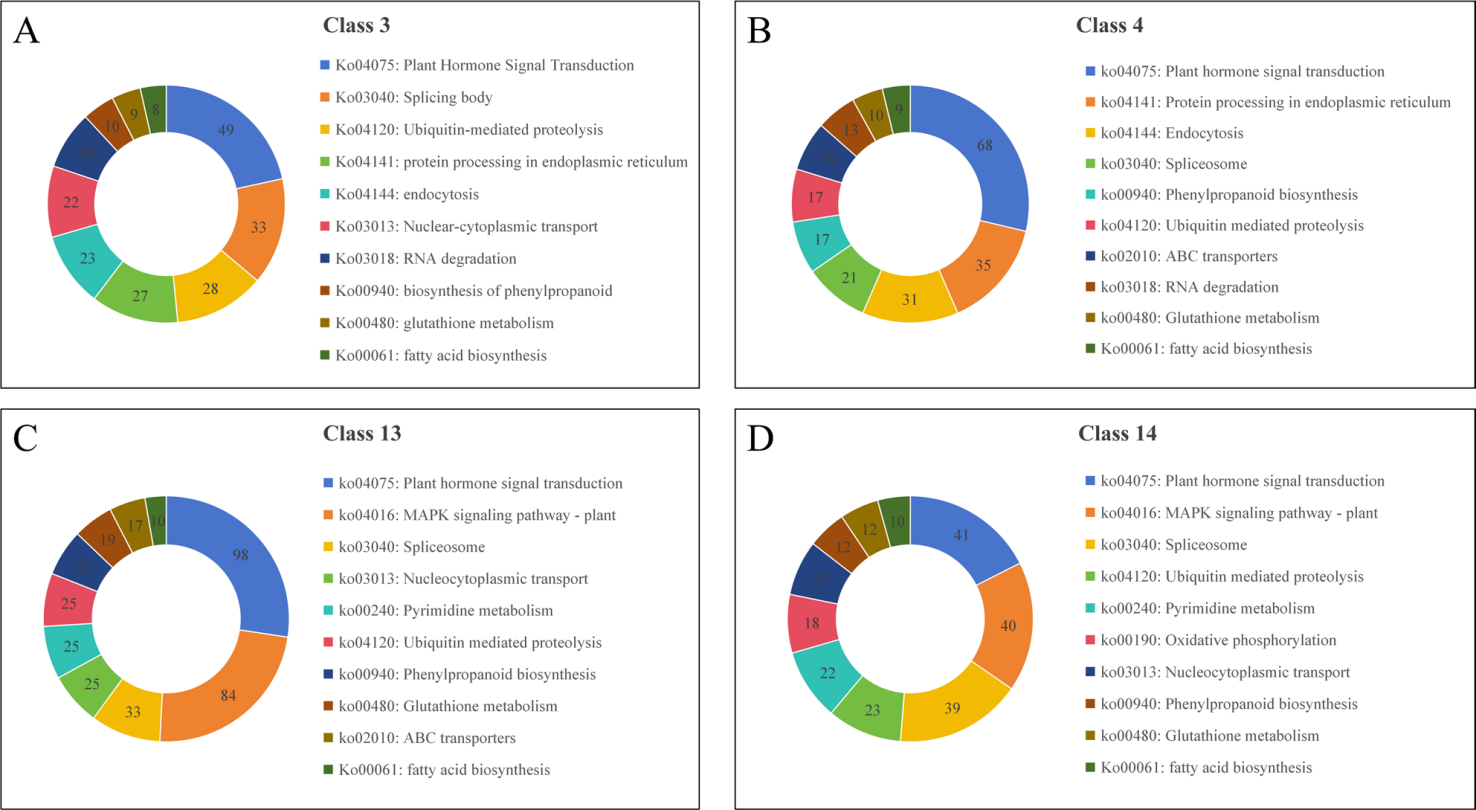
KEGG enrichment analysis of differentially expressed genes in each module. **A** Class 3. **B** Class 4. **C** Class 13. **D** Class 14

To systematically characterize the dynamic transcriptional responses to thermal exercise, we performed Mfuzz clustering analysis of all differentially expressed genes (DEGs) across treatment groups, identifying 16 distinct expression pattern clusters (Fig. 5). Compared with the UP group, the upregulated genes in the P3 group were significantly enriched in class 3, class 4, class 13 and class 14. After P1 treatment, the expression level of DEGs in class 3, class 4 and class 14 was up-regulated, while after P2 treatment, the expression level of DEGs in class 4 was down-regulated, but further up-regulated in class 3 and class 14. DEGs in class 13 was up-regulated only after P3 treatment. After KEGG enrichment analysis of DEGs in the above four classes, it was found that they were all enriched in plant hormone signal transduction, spliceosome pathway, phenylpropanoid biosynthesis pathway, glutathione metabolism pathway and fatty acid biosynthesis pathway (Fig. 6).

These results demonstrate that thermal exercise induces phase-dependent transcriptional reprogramming, with coordinated activation of stress-responsive metabolic and signaling pathways that collectively enhance alfalfa’s heat tolerance.

### Expression patterns of brassinosteroid-related DEGs

**Fig. 7:**
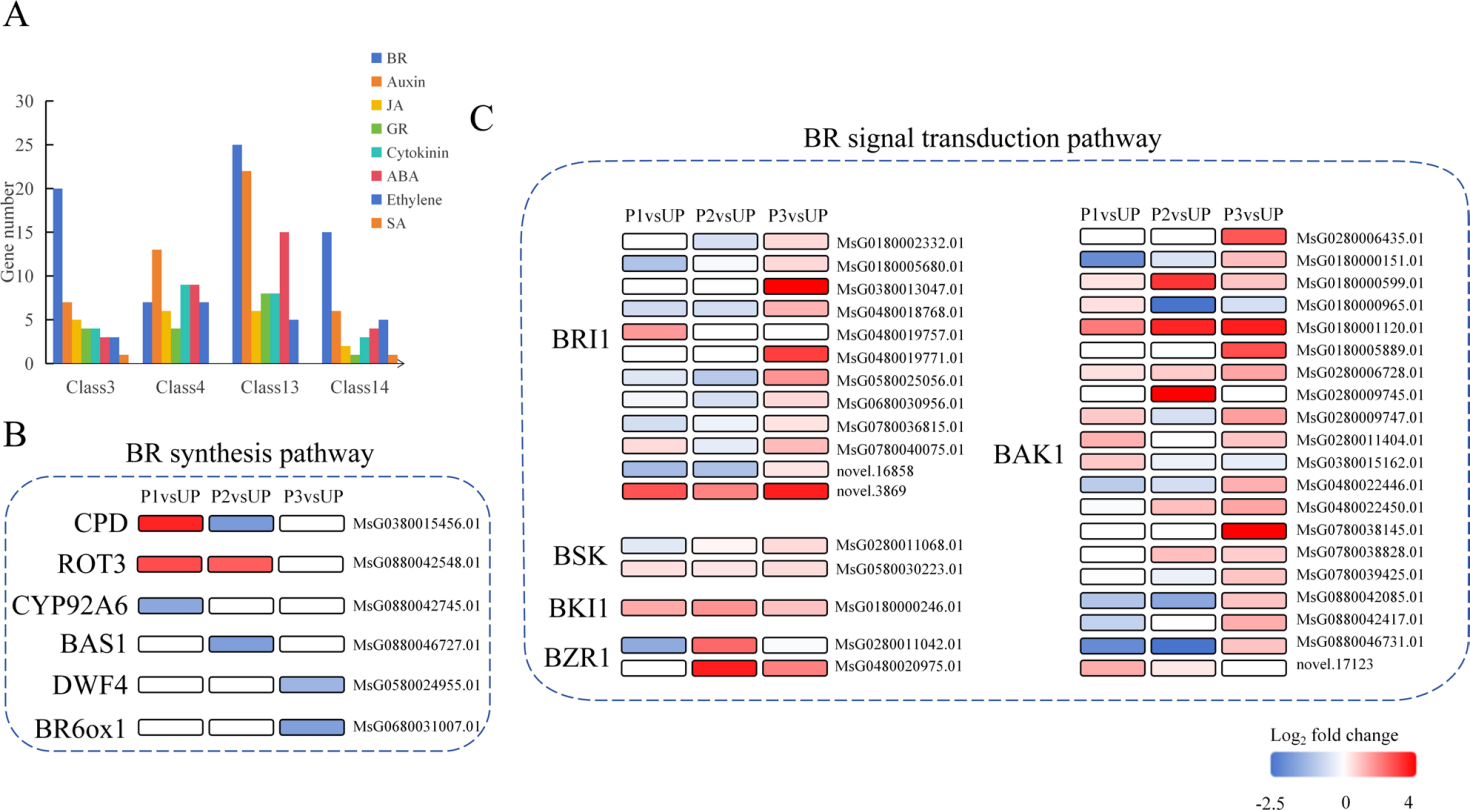
Effect of thermal priming on hormone signal transduction pathway. **A** Hormone signal statistics in each module. **B** Brassinolide synthesis pathway. **C** Brassinolide signal transduction pathway

Through classification and statistical analysis of “plant hormone signal transduction”-related genes in clusters 3, 4, 13, and 14, we observed significant enrichment of BR-related genes across all clusters (Fig. 7A). BRs are a class of steroid-structured phytohormones that play crucial roles in promoting plant growth and enhancing stress resistance to drought, high temperature, and low temperature conditions. Among BR compounds, brassinolide (BL) exhibits the highest biological activity. BL biosynthesis originates from campesterol (CR) through a series of enzymatic reactions (Fig. 7B). The BR receptor is the membrane-localized receptor-like protein kinase BRI1. BR binding to BRI1 triggers a phosphorylation cascade that activates downstream transcription factors (e.g., BES1/BZR1). These transcription factors subsequently translocate to the nucleus to regulate expression of target genes, thereby modulating plant growth, development, and stress responses [14, 15]. To elucidate the molecular regulatory mechanisms of BR in thermal priming responses, we systematically analyzed expression patterns of genes involved in BR biosynthesis and signaling pathways across different treatment groups. As illustrated in Fig. 7B, the BR biosynthetic pathway involves key enzymatic steps including sterol hydroxylation, side chain modification, and oxidation, mediated by genes encoding: sterol C-23 hydroxylase (CPD), sterol side chain modification enzyme (ROT3), cytochrome P450 family protein (CYP92A6), 2-Cys peroxidase (BAS1), dwarfing-related protein (DWF4), and C-6 oxidase (BR6ox1). Expression profiling revealed that while *CPD* and *ROT3* were upregulated following P1 treatment, other biosynthetic genes (including *DWF4* and *BR6ox1*) showed transcriptional suppression. Notably, despite downregulation of most BR biosynthetic genes after P1 treatment, key BR signaling components such as *BRI1*, *BAK1*, and *BZR1* were upregulated (Fig. 7C). These findings demonstrate that the BR-BRI1-BES1 signaling pathway plays a pivotal role in thermal priming-induced heat tolerance enhancement in alfalfa.

### Effect of thermal priming on spliceosome pathway

**Fig. 8:**
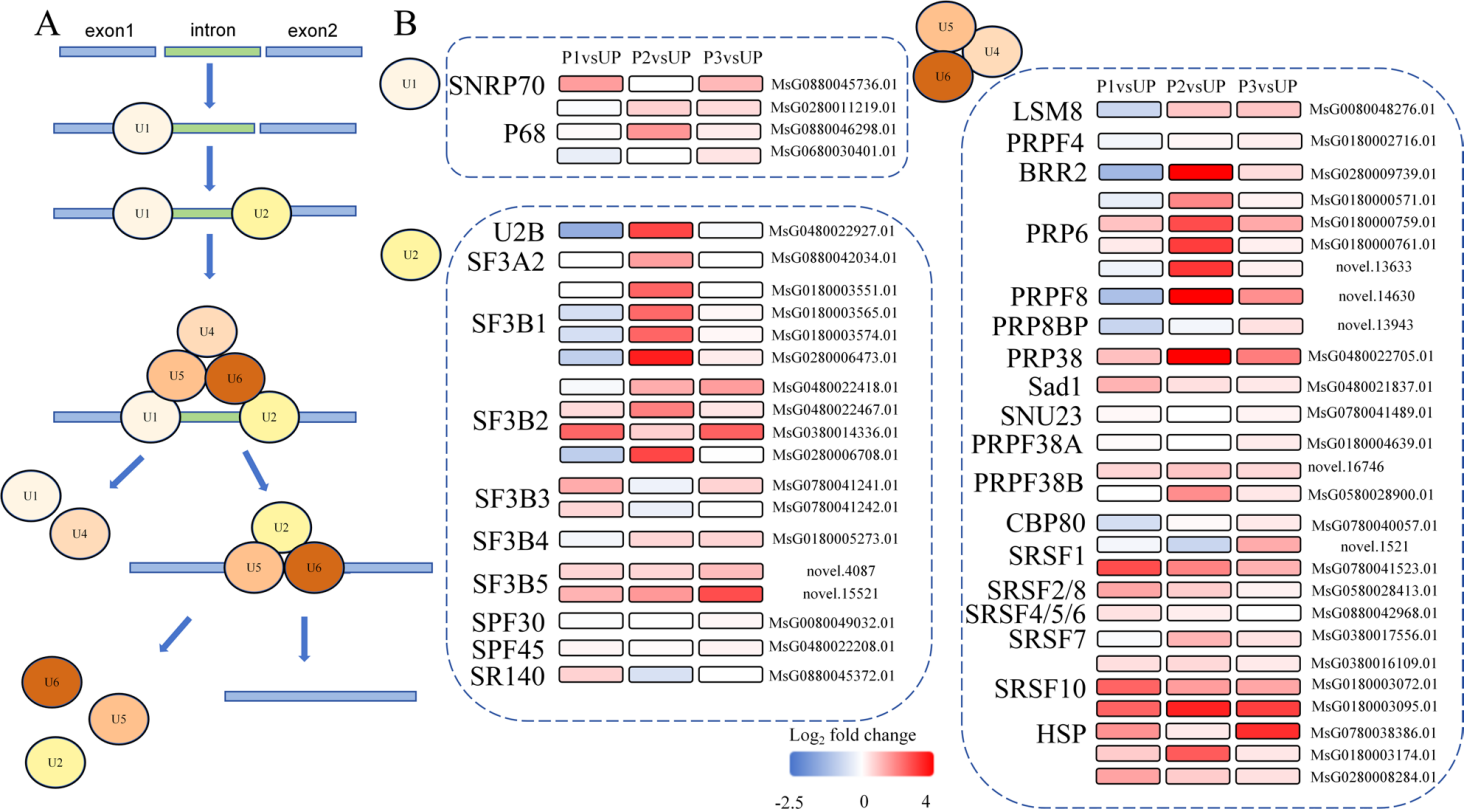
Effect of thermal priming on RNA splicing pathway. **A** Spliceosome pathway. **B** Gene expression of various components in the spliceosome pathway

The spliceosome pathway is a core mechanism in plant gene expression regulation, playing an irreplaceable role in plant growth, development, environmental adaptation, and response to external signals [16, 17]. To investigate the molecular regulatory mechanisms of the spliceosome pathway under thermal priming, we systematically analyzed the composition and expression patterns of spliceosome-related genes (Fig. 8A) across different groups (class 3, 4, 13, and 14). Expression profiling revealed upregulation of key spliceosome factors, including SNRP70 (involved in 5’ splice site recognition by U1 snRNP) and P68 (which unwinds the U1-5’ splice site duplex) (Fig. 8B). U2 snRNP is responsible for identifying the branch point sequence (BPS) within the intron region. U2B facilitates the anchoring of U2 snRNP to precursor mRNA via nonspecific binding of the splicing factor 3B(SF3B) complex to the BPS. As shown in Fig. 8B, both U2B and SF3B exhibited significant upregulation. Similarly, within the U4/U6. U5 tri-snRNP complex, genes associated with structural maintenance and mature mRNA splicing were markedly upregulated. These findings demonstrate that thermal priming induces significant upregulation of spliceosome pathway-related genes.

### Effect of thermal priming on phenylpropanoid biosynthesis pathway

**Fig. 9:**
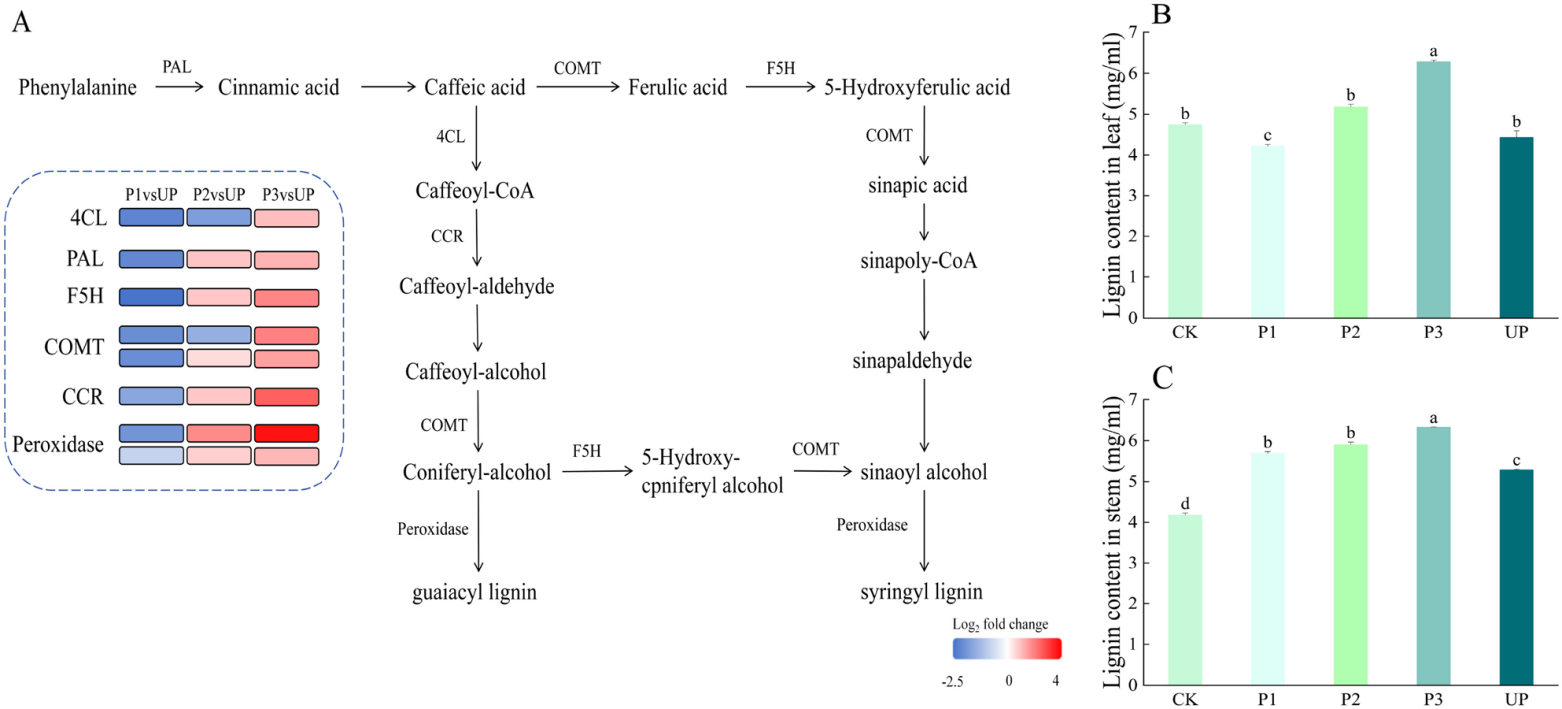
Effect of thermal priming on phenylpropanoid biosynthesis pathway. **A** Thermal map of lignin synthesis pathway. **B** Lignin content in leaves. **C** Lignin content in roots. Data were expressed as the mean ± standard error of three independent biological replicates. Different letters indicated significant differences of *P* < 0.05 according to Duncan test

Lignin, a key component of the secondary cell wall, provides structural rigidity and mechanical strength to plants, facilitating water transport and enhancing resistance to adverse environmental conditions [18]. KEGG enrichment analysis revealed significant upregulation of lignin biosynthesis-related genes following thermal exercise (Fig. 9A). The upregulation of key phenylpropanoid pathway genes may promote the accumulation of secondary metabolites, including phenols and lignin. Lignin content measurements in alfalfa leaves and stems demonstrated that the P3 group exhibited significantly higher lignin accumulation compared to the UP group. Under heat stress, both the UP and P3 groups showed increased lignin content relative to the CK group, with the P3 group displaying the highest levels (Figs. 9B-C). These findings indicate that thermal priming enhances lignin synthesis in alfalfa under heat stress, leading to greater stem lignin deposition, improved mechanical strength, and enhanced lodging resistance.

### Effect of thermal priming on glutathione metabolic pathway

**Fig. 10:**
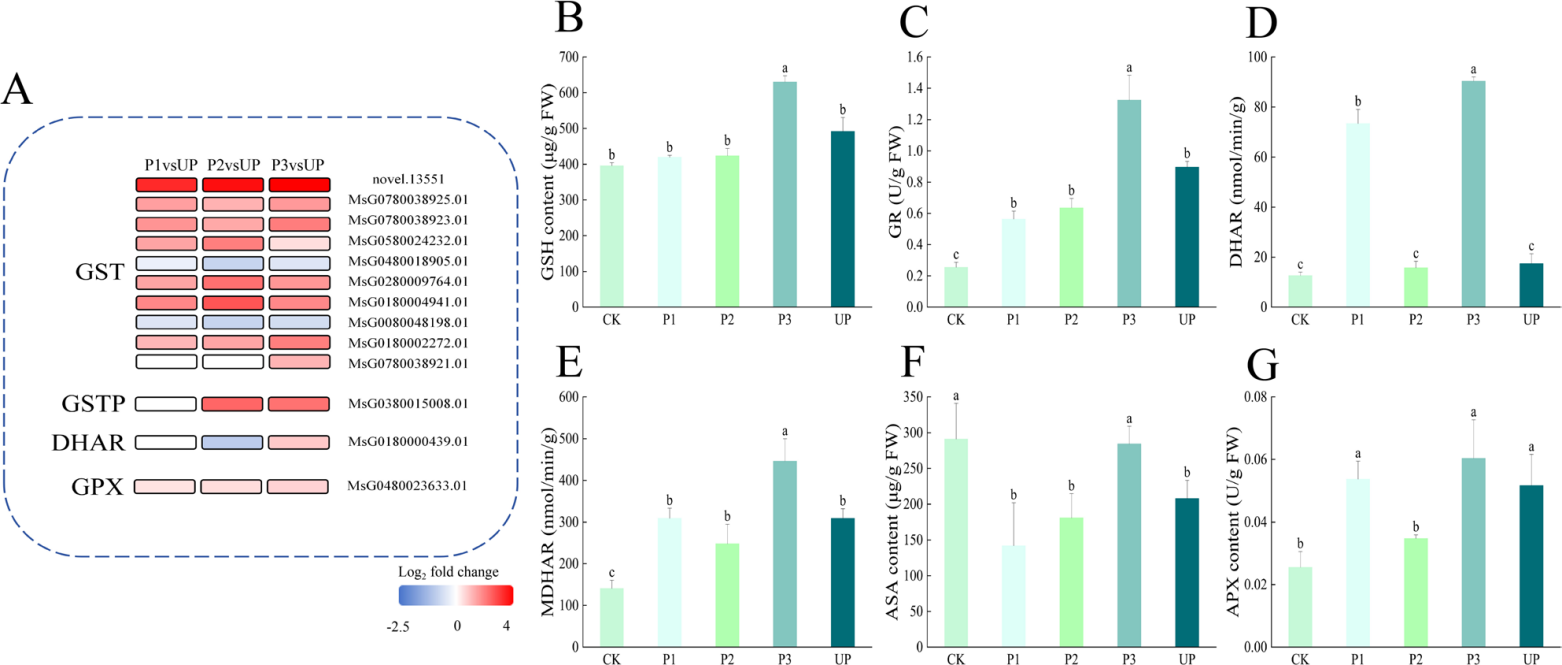
Effect of thermal priming on glutathione metabolism pathway. **A** Thermal map of glutathione metabolic pathway. **B** GSH content. **C** GR activity. **D** DHAR activity. **E** MDHAR activity. **F** AsA content. **G** APX activity. Data were expressed as the mean ± standard error of three independent biological replicates. Different letters indicated significant differences of *P* < 0.05 according to Duncan test

The glutathione metabolic pathway plays a crucial role in plant stress responses, with glutathione S-transferase (GST) serving as the key enzyme that catalyzes the initial step of glutathione (GSH) conjugation [19]. As a vital antioxidant, glutathione exhibits both antioxidant and detoxification properties. Furthermore, it participates in the AsA-GSH cycle, forming an essential component of the non-enzymatic antioxidant system and representing a primary pathway for ROS scavenging in plants. Enrichment analysis revealed significant upregulation of genes involved in glutathione metabolism, including GST and glutathione peroxidase (GPX), following thermal exercise. As shown in Fig. 10A, antioxidant-related genes such as *GST* and *GPX* are rapidly activated in P1 stage of hot start. Dehydroascorbic acid reductase (DHAR) and glutathione-S-transferase Pi (GSTP) were activated in the following P2 and P3 stages. This immediate response suggests that alfalfa rapidly initiates its antioxidant defense mechanisms to counteract heat stress-induced damage. Notably, the expression of these antioxidant enzyme genes further increased after heat stress, indicating that thermal exercise not only triggers the initial antioxidant response but also enhances the plant’s capacity to withstand subsequent heat stress.

Comparative analysis of biochemical components showed that under heat stress conditions, both UP and P3 groups exhibited increased levels of GSH, glutathione reductase (GR), DHAR, monodehydroascorbate reductase (MDHAR), and ascorbate peroxidase (APX) compared to the CK group, while demonstrating lower AsA content. Importantly, the P3 group showed higher concentrations of GSH, GR, DHAR, MDHAR, AsA, and APX than the UP group (Figs. 10B-G). These findings demonstrate that thermal priming induces the expression of *GST*, *DHAR*, and related genes, leading to increased accumulation of antioxidants including GSH and APX. This response promotes efficient operation of the AsA-GSH cycle, enhances overall antioxidant capacity, and consequently improves alfalfa’s thermotolerance when facing subsequent high-temperature stress.

### Effect of thermal priming on fatty acid composition and content

Fatty acids serve as fundamental components of cell membranes, where their saturation degree and carbon chain length critically influence membrane fluidity. These properties are essential for plant growth, physiological metabolism, and abiotic stress resistance [20]. To investigate whether thermal priming enhances plant thermotolerance through fatty acid (FA) modulation, we analyzed free fatty acid profiles using gas chromatography-tandem mass spectrometry (GC-MS). Our analysis identified 17 free fatty acids across P1, P2, P3, UP, and CK groups (Table S3). The composition revealed that palmitic acid (6.7%−11% of total FAs) was predominated in saturated fatty acids (SFA), followed by stearic acid (1.3%−2.3%). Among the monounsaturated fatty acids (MUFA), oleic acid had the highest content, followed by erucic acid, accounting for 1.7%−4.1% of total fatty acids. Among polyunsaturated fatty acids (PUFA), the highest content was α-linolenic acid, accounting for 74.6%−80.6% of total fatty acids, followed by linoleic acid, accounting for 5.3%−8.1% of total fatty acids. Notably, the P3 group exhibited significantly higher total FA content (600.52 ± 61.70 µg/g) versus control (402.52 ± 2.73 µg/g), P1 (448.11 ± 9.58 µg/g), P2 (561.69 ± 49.66 µg/g), and UP (424.52 ± 19.62 µg/g) groups.

**Fig. 11:**
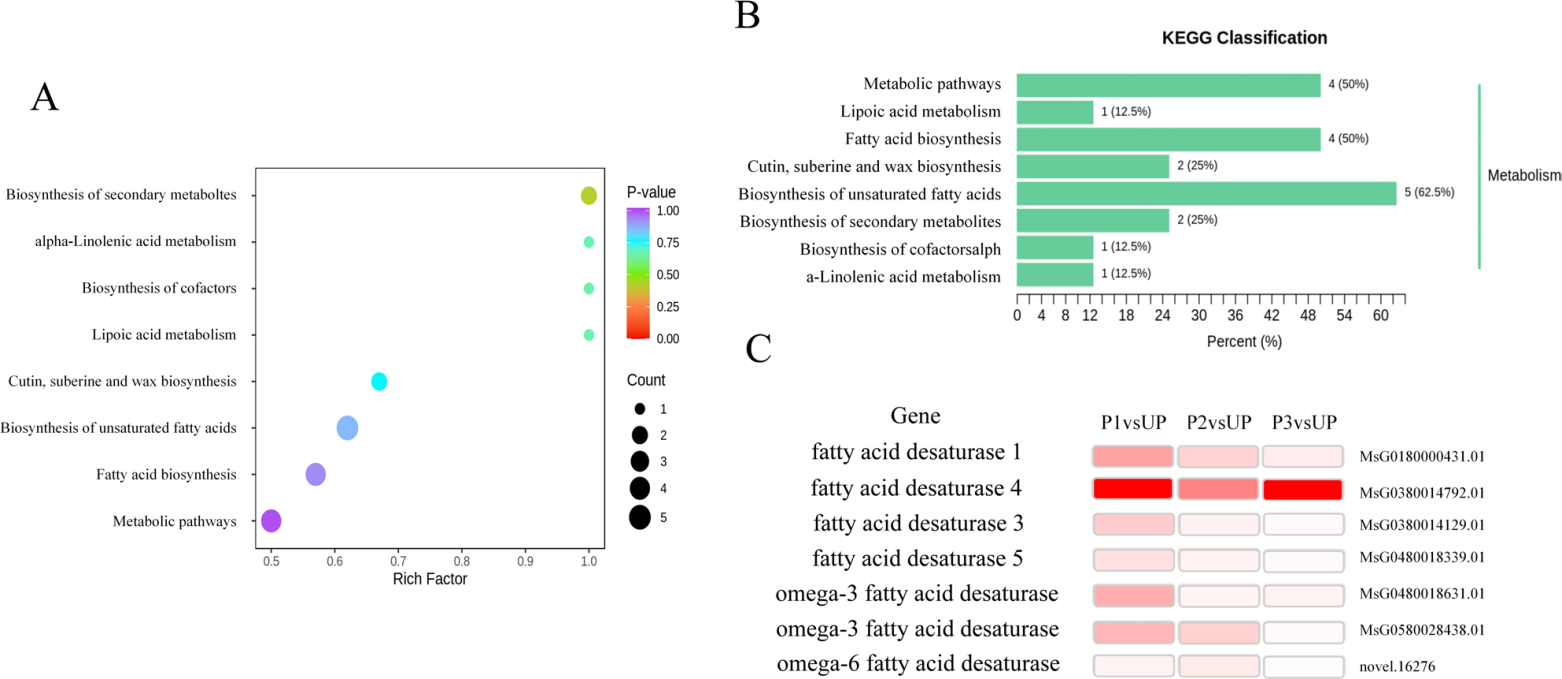
Effect of thermal priming on fatty acid content. **A** Dotplot of KEGG enrichment analysis of differential metabolites. **B** Barplot of KEGG enrichment analysis of differential metabolites. **C** Thermal map of fatty acid desaturase gene expression

KEGG enrichment analysis of P3 vs UP metabolites showed significant enrichment in unsaturated fatty acid biosynthesis, metabolic pathways, and fatty acid biosynthesis (Figs. 11A-B). Transcriptional data further indicated upregulation of *FAD* genes across all groups. According to |log2FC| >0.6, *P* < 0.05, the differential metabolites in P3 vs UP group were screened (Table 1). The main differential metabolites are α-linolenic acid, erucic acid, oleic acid, arachidic acid and docosanoic acid. Among unsaturated fatty acids, the contents of α-linolenic acid, oleic acid and erucic acid in P3 group increased by 52.6%, 147.1% and 107.5% respectively compared with those in UP group, showing an upward trend. Among linear saturated fatty acids, arachidonic acid and docosanoic acid in P3 group were 52.5% and 51.6% of those in UP group, respectively, showing a downward trend. While SFA levels remained stable, MUFAs and PUFAs increased markedly, particularly PUFAs. This suggests thermal priming preferentially enhances unsaturated fatty acid synthesis, potentially improving membrane fluidity and heat resistance in alfalfa.

**Table 1:** The content of different fatty acids in P3 vs UP group

Fatty acid name	Type	P3 (µg/g)	UP (µg/g)	*P*-value	log2FC	Type
α-linolenic acid	Polyunsaturated fatty acid	483.783 ± 49.726a	317.118 ± 1.437c	0.022	0.609	UP
erucic acid	Monounsaturated fatty acids	0.944 ± 0.008	0.455 ± 0.027	0.018	1.053	UP
oleic acid	Monounsaturated fatty acids	23.972 ± 3.315	9.696 ± 3.315	0.014	1.305	UP
arachidic acid	Linear saturated fatty acid	0.426 ± 0.026	0.811 ± 0.101	0.014	−0.929	DOWN
docosanoic acid	Linear saturated fatty acid	0.304 ± 0.053	0.589 ± 0.031	0.049	−0.954	DOWN

### Validation of RNA-seq data by quantitative RT-PCR (RT-qPCR)

**Fig. 12:**
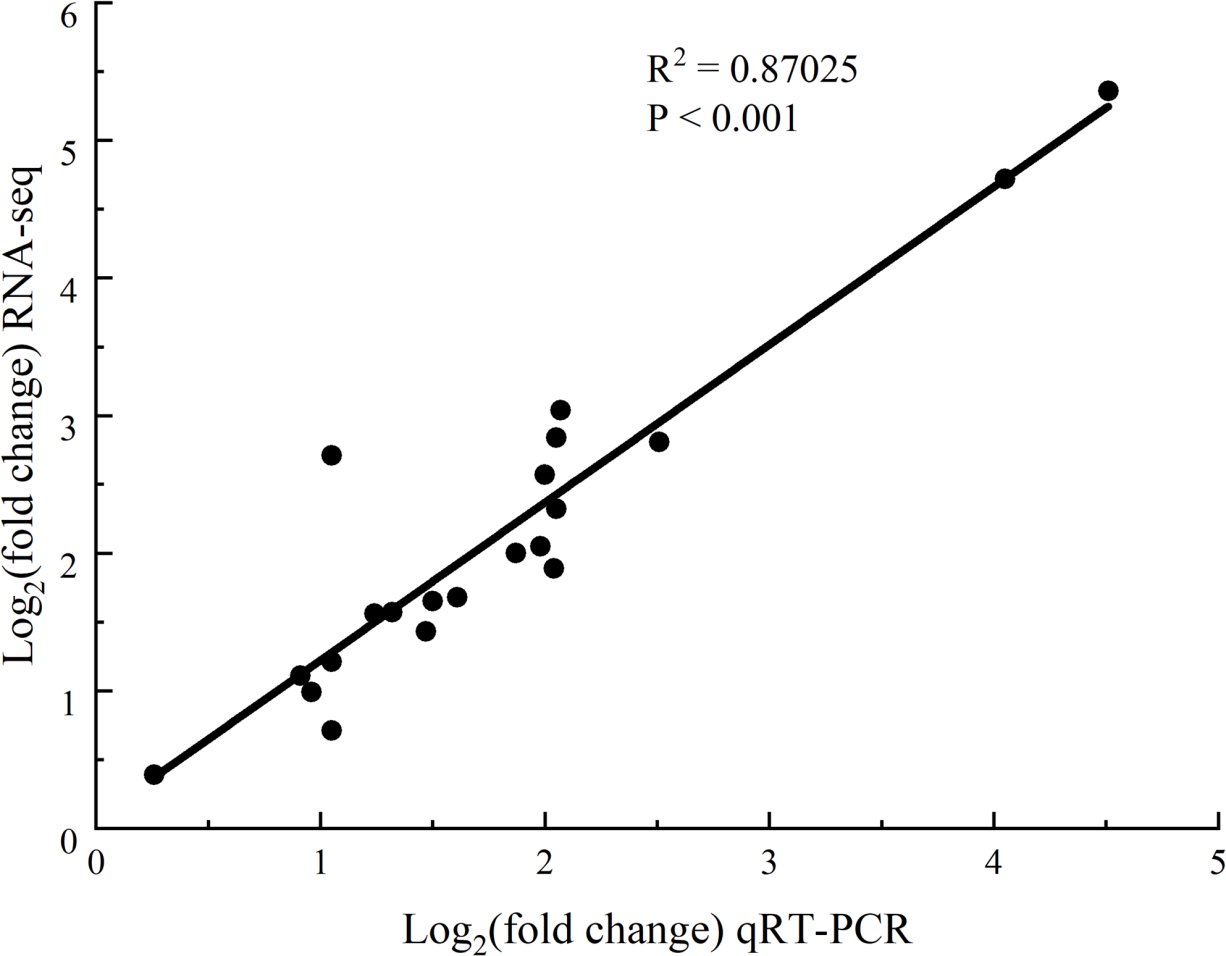
Validation of gene expression by RT-qPCR analysis

To validate the reliability of our RNA-Seq results, we selected seven key genes involved in phenylpropanoid biosynthesis and glutathione metabolism pathways for RT-qPCR analysis. The RT-qPCR results demonstrated excellent consistency with the RNA-Seq data (Fig. 12), confirming the accuracy of our transcriptome sequencing findings. This strong correlation between both analytical methods validates the differential gene expression patterns observed in response to thermal exercise.

## Discussion

As a critical physiological response mechanism to environmental temperature fluctuations, thermal priming enables plants to systematically adjust their physiological processes following exposure to high temperatures, thereby enhancing their capacity to withstand subsequent heat stress. This adaptive response exhibits temporal persistence, as plants retain elevated thermotolerance even after ambient temperatures normalize. The underlying mechanisms involve complex biochemical and molecular reprogramming, including gene expression modulation, metabolic pathway adjustments, and signal transduction activation.

### Oxidative stress and antioxidant defense

Heat stress disrupts cellular redox homeostasis, leading to ROS accumulation. While ROS function as signaling molecules regulating growth and stress responses [21], excessive ROS induces oxidative damage to DNA, proteins, and lipids, ultimately impairing cell viability [22]. Plants counteract ROS via enzymatic (SOD, POD, CAT) and non-enzymatic (GSH, AsA) antioxidant systems [23]. In this study, heat-stressed alfalfa exhibited increased SOD, POD, and CAT activities. Notably, thermally primed (P3) seedlings displayed further elevation in SOD and CAT activities but reduced POD activity compared to unprimed (UP) plants, suggesting that thermal priming preferentially scavenges superoxide radicals (O^2−^) and H₂O₂, thereby diminishing POD demand. Consequently, P3 seedlings maintained lower ROS levels and superior growth under heat stress, underscoring the role of thermal priming in enhancing oxidative stress resilience.

### Brassinosteroid (BR) signaling and heat tolerance

As a key phytohormone, BR enhances plant thermotolerance. Prior studies demonstrate that BR elevates photosynthetic efficiency (Fv/Fm) and stomatal conductance in heat-stressed rice [24]. BR activates the expression of *HSFA2*, *DREB2A*, and *HSP*s via BZR1-mediated repression of ERF49 in *Arabidopsis* [25]. In addition, BR promotes dry matter and nutrient translocation in maize under heat stress [26]. BR regulates HSP17.6 A via histone acetyltransferase HAC1 to sustain proteostasis [27]. Our transcriptomic analysis revealed that thermal priming potentiates BR signaling by upregulating receptor genes (*BRI1*/*BAK1*). The transcription factor BZR1, a central BR pathway regulator, was upregulated, directly activating downstream thermotolerance genes, including HSPs and genes involved in mitigating ROS toxicity.

BR signaling pathway can regulate several downstream metabolic pathways and secondary metabolic pathways. Liu et al. found that BR treatment increased the activities of PAL and 4CL in watermelon seedlings under zinc stress and induced lignin accumulation [28]. Under salt stress, the contents of phenolic compounds, flavonoids and lignin were increased by BR in order to reduce the damage in Ornamental Gourd [29]. Guo et al. found that BR can increase lignin synthesis-related genes such as *PAL* and lignin deposition in *Ginkgo biloba* [30]. In Korean pine, BR can also improve PAL activity and lignin content in cells through phenylpropanoid biosynthesis [31]. Zhou et al. found that BR in grapes can enhance AsA-GSH cycle by increasing the activities of MDHAR, GR, APX and DHAR, and the contents of antioxidant ascorbic acid (MA) and dehydroascorbic acid (DHA), thus reducing the damage of plants under Cu stress [32]. Niu et al. found that under low temperature stress, BR promoted the accumulation of phenols such as GSH and hesperidin in jujube fruit, maintained the quality and reduced chilling injury [33]. Dong et al. found that BR treatment can significantly up-regulate genes such as *FAD2*, *FAD3* and *LOX* in grapes, maintain the proportion of unsaturated fatty acids in membrane lipids, and realize the stability of membrane structure [34]. Li et al. found that BR can also increase the accumulation of butyric acid, octanoic acid, decanoic acid, linoleic acid and other substances in grapes, and improve their low temperature resistance [35]. In this study, thermal priming activated these pathways, increasing metabolites (e.g., lignin, phenolics, unsaturated fatty acids) that collectively bolster heat tolerance in alfalfa.

### Phenylpropanoid pathway and lignin deposition

Phenylpropanoids (e.g., flavonoids, lignin) are pivotal for stress adaptation [36]. Phenylpropionic acids are mainly derivatives of cinnamic acid, including caffeic acid, ferulic acid, mustard acid, etc. Because of their excellent ability of scavenging free radicals, they are considered as the main antioxidants to resist oxidative damage and are necessary for plants to adapt to biotic and abiotic stresses [37]. Lignin is one of the important products in the biosynthesis of phenylpropanoid. It can not only enhance the mechanical strength of plants and the hardness of cell walls but also have many biological functions such as resisting the invasion of adverse external environment and diverting water transport in tissues [38]. Lignin is synthesized by phenylpropanoid biosynthesis with phenylalanine as the substrate. PAL converts phenylalanine into cinnamic acid, which is then reduced by 4CL, CCR, CAD and other enzymes in turn to generate corresponding coenzyme A, and finally the catalytic intermediate coenzyme A is converted into corresponding lignin monomer [39]. Under heat stress, phenylpropanoids are considered as markers of heat stress in plants [40]. For example, phenylpropionic acids and flavonoids in carrot cells protect plants from heat stress by inhibiting ROS formation [41]. Paupière et al. found that HSFb1 induced the accumulation of phenylpropanoid metabolites in tomatoes, which enhanced the heat resistance [42]. In this study, the expression levels of PAL, COMT, F5H, CCR and 4CL related to lignin biosynthesis increased significantly after thermal priming. Compared with CK, it was observed that the expression of lignin synthesis-related genes decreased in the early stage of thermal exercise, which may be because high temperature interfered with enzyme activity and transcription factor binding ability, thus inhibiting the normal expression of related genes. At P3 stage after thermal priming, the expression of lignin synthesis-related genes was significantly higher than that of UP group without thermal priming. This shows that through thermal priming, plants may start the adaptive mechanism, enhance their structural stability and stress resistance, and up-regulate the expression of lignin synthesis-related genes, thus promoting lignin synthesis and improving the resistance to high temperature. The quantitative results of lignin content showed that the lignin content in alfalfa stems increased, which is also the reason why plants after thermal priming showed stronger lodging resistance. In addition, the determination of lignin content in seedling stems of P1 and P2 treatment points during thermal priming showed that alfalfa began to synthesize lignin at the early stage of thermal priming and accumulated continuously. In a word, thermal priming promotes the synthesis and accumulation of lignin in alfalfa stems and effectively improves the lodging resistance of alfalfa seedlings.

### AsA-GSH cycle and redox balance

The ascorbate-glutathione (AsA-GSH) cycle represents a crucial antioxidant defense system in plants, functioning to scavenge ROS through coordinated action of antioxidants (AsA and GSH) and enzymes including APX, GPX, and glutathione reductase (GR) [43]. In this cycle, APX catalyzes the oxidation of AsA to eliminate harmful ROS such as superoxide radicals (O^2−^) and hydrogen peroxide (H₂O₂), while GR, DHAR, and MDHAR regenerate reduced AsA and GSH to sustain the cycle’s activity [44]. Extensive research has established a positive correlation between AsA-GSH cycle activity and plant stress tolerance. For instance, strawberry seedlings exposed to combined high temperature and high light stress exhibited enhanced GR, DHAR, and MDHAR activities, facilitating AsA-GSH regeneration and consequently improving stress resilience [45]. Similarly, Li et al. demonstrated that the halophyte *Suaeda salsa* upregulates GR under salt stress to boost GSH production and alleviate oxidative damage [46]. Our experimental findings demonstrate that thermal priming significantly enhances the AsA-GSH cycle’s efficiency in alfalfa seedlings. Quantitative analyses revealed elevated levels of both enzymatic components (APX, GR, DHAR, MDHAR) and non-enzymatic antioxidants (AsA, GSH) in primed plants. This coordinated upregulation enables more effective ROS detoxification and redox homeostasis maintenance, thereby reducing oxidative stress damage under high temperature conditions. The enhanced AsA-GSH cycle activity represents a key mechanism through which thermal priming confers improved thermotolerance in alfalfa seedlings.

### Fatty acid remodeling and membrane stability 

As a key component of biofilm, cork and plant epidermis wax, fatty acids are not only the basic raw materials for building cell and tissue structures, but also effectively maintain the material exchange regulation and barrier function between organisms and the external environment by forming hydrophobic barriers, and also play an important role in resisting abiotic stresses [47]. At higher temperature, plants reduce the phase transition temperature by increasing unsaturated fatty acids in membrane lipids, to keep the cell membrane liquid-crystalline at high temperature and avoid the loss of cell function caused by the solidification or rupture of membrane structure, and thus alleviate the influence of high temperature stress. For example, the deletion of C18:1 and C18:2 in *accD-C794* mutant in *Arabidopsis thaliana* leads to heat sensitivity, which indicates that unsaturated fatty acids play an important role in heat stress tolerance [48]. This ability mainly depends on the regulation of fatty acid desaturase (FAD) on unsaturated fatty acid level [49]. Beisson et al. found that in *Arabidopsis thaliana*, the mutant with *FAD3* gene deletion showed serious membrane damage at 42℃, and the linolenic acid content in chloroplast membrane lipid decreased by 60%, accompanied by a three-fold increase in MDA accumulation [50]. Chen et al. found that under the continuous stress of 38℃, the leaf EL of the rice *OsFAD3* overexpression line decreased by 45% compared with that of the wild type, indicating that the membrane permeability was effectively maintained [51]. Our transcriptome analysis showed that thermal priming increases the expression of *FAD* gene, causes the accumulation of polyunsaturated fatty acids and maintains the stability of membrane system.

High temperature changes of membrane lipid from liquid crystal state to gel state, which destroys the membrane structure. The three double bonds of α-linolenic acid (C18:3) make the membrane have high fluidity, which can reduce the phase transition temperature of the membrane and delay the curing of membrane lipid induced by high temperature [52]. You et al. found that the decrease of α-linolenic acid content in rice would destroy the integrity and stability of cell membrane system under heat stress [53]. Ibrahim et al. found that after tomato seeds were soaked at 50℃ for 2 h, the α-linolenic acid content increased, and the germination ability was significantly improved [54]. Tang et al. found that α-linolenic acid can also produce jasmonic acid (JA) through lipoxygenase (LOX) pathway and then activate the expression of *HSFs* and *HSPs* through JA, thus alleviating high temperature injury [55]. In this study, the content of α-linolenic acid was the highest in P3 group, which was significantly increased by 52.6% compared with UP group. The results showed that thermal priming could reduce the injury of alfalfa seedlings at high temperature by increasing the content of α-linolenic acid. As a monounsaturated fatty acid, the accumulation of oleic acid may improve the thermal stability by lowering the lipid transformation temperature of the membrane. Hou et al. found that soybean seeds increased oleic acid content at high temperature as an adaptation mechanism [56]. Li et al. found that in soybean *GmPDCT1*/*GmPDCT2* silent mutant, the transformation from oleic acid to linoleic acid was blocked, and the SOD activity and POD activity of leaves increased by 45% and 60% respectively under salt stress [57]. In this study, the oleic acid content of P3 group increased by 147.1% compared with that of UP group, which indicates its important role in improving the heat resistance of alfalfa under high temperature stress. The synthesis of erucic acid (C22:1) is based on oleic acid-CoA. Through continuous condensation, reduction, dehydration and reduction, two carbon units are added each time, and erucic acid is generated after three rounds of extension. As a long-chain monounsaturated fatty acid, erucic acid may help plants maintain the stability of membrane structure under high temperature stress by changing the fluidity of cell membrane lipids. There was no significant difference in saturated fatty acid content between P3 group and CK group, but it was significantly lower than P2 group. It is speculated that the membrane fluidity can be optimized by increasing the content of saturated fatty acids and balancing the ratio of saturated and unsaturated fatty acids in alfalfa during thermal exercise to avoid excessive liquefaction of the membrane at high temperature.

### Spliceosome activation and transcriptional plasticity

Spliceosome are dynamic complexes composed of snRNP (such as U1, U2, U4/U6, U5) and non-snRNP proteins. Spliceosome pathway is not only a molecular switch of gene expression in plants, but also a key hub connecting genetic information and phenotypic plasticity. By precisely regulating alternative splicing and constitutive splicing, it gives plants molecular flexibility to cope with complex environments [58]. For example, under low temperature stress, small nuclear ribonucleoprotein E1(SME1) ensures the correct splicing of many mRNA precursors in *Arabidopsis thaliana* [59]. In addition, He et al. found that under heat stress, the activity and composition of splicing also change significantly [60]. Gu et al. found that the spliceosome pathway in Chinese cabbage was significantly up-regulated under high temperature stress [61]. Lee et al. found that splicing factor 1(SF1) participated in the regulation of flowering and heat tolerance by participating in the splicing of the precursor of HsfA2 [62]. Rosenkranz et al. found that five Serine/Arginine-rich(*SR*) genes in tomato were up-regulated by high temperature induction to optimize transcription and splicing efficiency [63]. In this study, SNRP70 which recognizes the 5’ splicing site on the precursor mRNA in U1 snRNP, P68 which unwinds the duplex of U1-5’ splicing site, and Y2B, SF3B, Serum Response Factor(SRF) and other proteins which recognize and bind the branching sequence in U2 snRNP can make more U1 bind to the 5’ splicing site faster, and U2 bind to the branching point faster, thus accelerating the early assembly of splices and allowing cells to process more precursor mRNA in a unit time. Up-regulation of key genes in U4/U6. U5 tri-snRNP complex makes the supply of the protein complex more abundant, which can accelerate the transformation of splice from “pre-splice” to “mature splice” and push splicing into the catalytic stage. In this study, after thermal priming, these spliceosome pathway-related genes were up-regulated rapidly after P1 treatment, which significantly increased the output rate of mature mRNA, and many proteins with different functions were synthesized rapidly. The rapid response of this post-transcriptional regulation mechanism is the key for plants to obtain high temperature adaptability during thermal priming. After heat-priming, the splicing process of precursor mRNA is accelerated by pre-activating the genes related to the spliceosome pathway, so that mature mRNA and functional protein can be synthesized rapidly.

## Conclusion

**Fig. 13:**
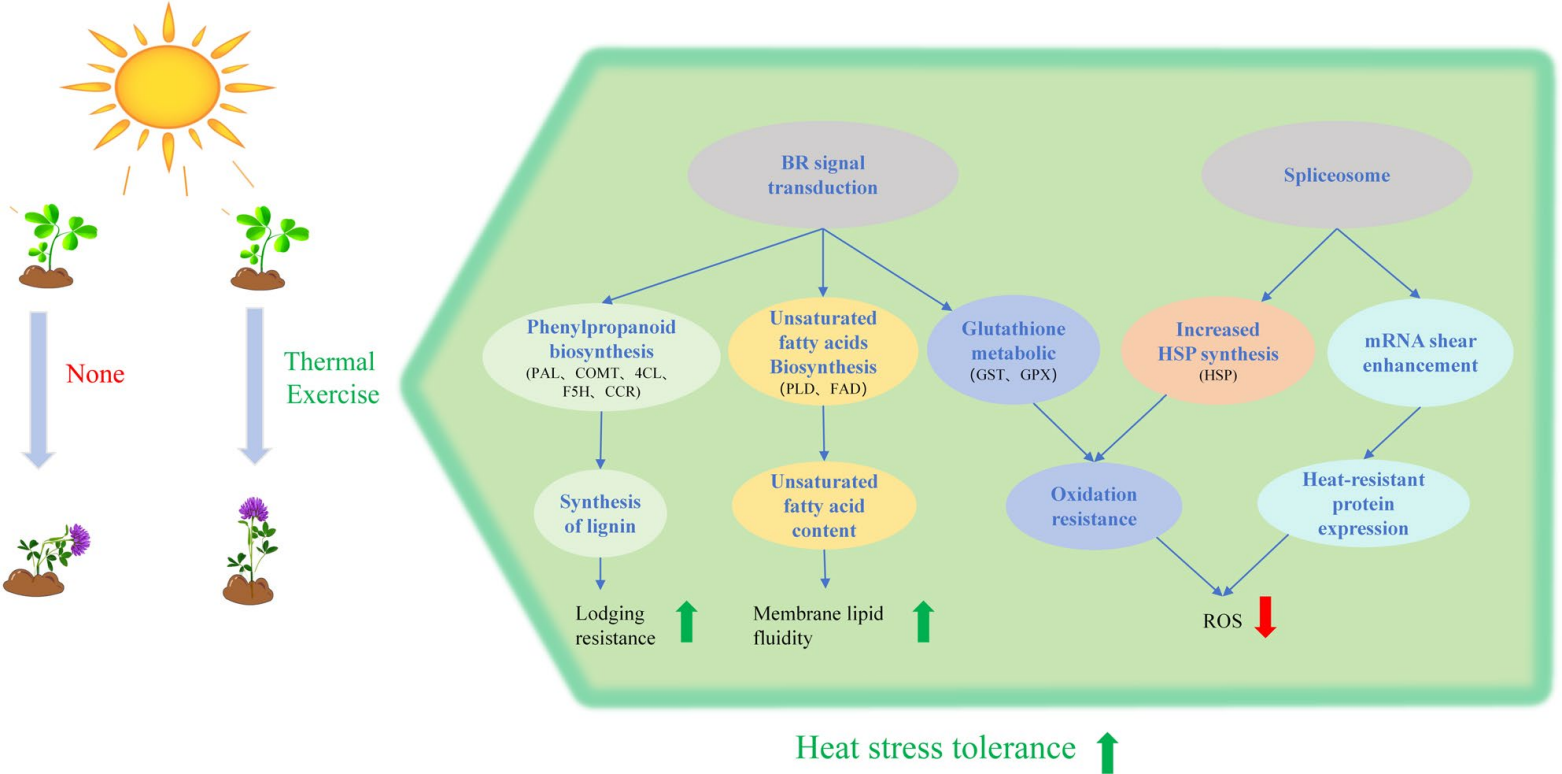
Hypothetical model of improving heat tolerance of alfalfa by thermal exercise. The green arrow indicates upward adjustment, and the red arrow indicates downward adjustment

This study demonstrates that heat priming enhances alfalfa’s thermotolerance by elevating chlorophyll content, soluble protein levels, and the activities of SOD and CAT, while reducing ROS accumulation and MDA content under high-temperature stress. Transcriptome analysis revealed that differentially expressed genes in P3 vs UP were significantly enriched in hormone signaling pathways, primary metabolite biosynthesis, and secondary metabolite biosynthesis, with a strong association with BR signaling. Mechanistically, heat priming exerts its protective effects through BR-mediated metabolic regulation and the phenylpropanoid biosynthesis pathway, leading to increased lignin synthesis in stems, thereby improving lodging resistance. Heat priming also boosts the AsA-GSH cycle, elevating antioxidant enzyme activity and non-enzymatic antioxidant levels to mitigate ROS-induced damage, and promotes unsaturated fatty acid synthesis, maintaining membrane fluidity under heat stress. On the other hand, heat priming facilitates mRNA splicing and export by upregulating the spliceosome pathway, ensuring efficient gene expression under stress. Through these synergistic mechanisms (Fig. 13), heat priming enables alfalfa to establish a robust thermotolerance defense system, ultimately enhancing its heat resistance.

## Materials and methods

### Plant materials and growing conditions

The experiment was conducted in the College of Environment and Resources of Dalian Minzu University in May 2023. Twelve commercial alfalfa varieties (Table S4) were purchased as experimental materials, and the seeds were soaked in the dark for 24 h to accelerate germination. Seeding the germinated seeds into flowerpots (10 cm×10 cm×10 cm), wherein the substrate is peat soil, vermiculite and pearl salt, which are stirred evenly at 2:1:1, and each pot is seeded with 8–10 alfalfa seeds, and each variety is planted in 10 pots, and then cultured in a solar greenhouse (average temperature 25.0 ± 2℃, relative humidity 60 ± 5%). During the cultivation period, water was given every 3 days to keep the soil moisture sufficient. After 14 days at three-leaf stage, alfalfa seedlings with similar growth and no diseases and pests were selected for the next experiment.

### Heat-resistance screening

By simulating the high temperature environment in an incubator, leaves of 12 alfalfa varieties under high temperature stress were collected to determine physiological and biochemical indexes, and the heat resistance of different alfalfa varieties was comprehensively evaluated by membership function method [64]. After 14 days of growth, alfalfa plants were moved to a constant temperature light incubator. The control group was cultured at 25℃, and the experimental group was treated with heat stress at 37℃. After 4 h treatment, leaves of alfalfa seedlings under heat stress (HT) and control (CK) were collected for determination of chlorophyll content, antioxidant enzyme activity, electrolyte permeability and MDA content. There were three independent plants in each of the three biological replicates for all the above experiments. Finally, the relative heat tolerance of alfalfa varieties was evaluated by membership function method.

### Thermal priming treatment

Heat-resistant alfalfa ‘Sanditi’ was used as the experimental material. After 12 days of cultivation, it was treated with high temperature stress as shown in Fig. 2.A, and it was divided into control group CK (Control), thermal exercise group P (Primed) and heat control group UP (unprimed) without heat priming. The plants were subjected to heat stress in a light incubator. The light intensity was 12,000 lx, the light period was 16 h light/8 h darkness, and the relative humidity was 60%. Pots were randomly placed to reduce the influence of different positions. In the thermal exercise group, P first exercised at 37℃ for 2 h, then recovered at 23℃ for 2 h, then exercised at 43℃ for 2 h, and then recovered at 23℃ for 2 days. Finally, P group and UP group were subjected to heat stress at 43℃ for 4 h. Leaf samples were collected from the four experimental groups: P1, P2, P3, CK and UP and were frozen with liquid nitrogen and stored in a deep freezer at −80℃ for subsequent experiments.

### Physiological index determination

Chlorophyll content in alfalfa leaves was determined by acetone-ethanol mixed extraction [65]. The content of MDA was determined by thiobarbituric acid colorimetry [66]. The activity of SOD was determined by nitroblue tetrazole photochemical reduction method [67]. The activity of POD was determined by guaiacol method [68]. The activity of CAT was determined by ultraviolet absorption method [69]. EL was measured by conductivity meter [70]. Coomassie brilliant blue method was used to determine the content of soluble protein. The leaves were infiltrated with NBT, DAB [71] and EB [72] respectively, and the histochemical staining was carried out to detect the accumulation of superoxide anion, hydrogen peroxide and cell death in alfalfa leaves. There were three independent plants in each of the three biological replicates for all the above experiments.

### Transcriptome sequencing and data analysis

A total of 15 samples (5 treatments×3 biological replicates) were collected for Qualcomm quantitative transcriptome sequencing detection. The experimental process included RNA extraction and detection, cDNA library construction and computer sequencing [73]. The total RNA of alfalfa leaves was extracted by Tiangen RNAprep pure plant kit (centrifugal column), and the applied methods and steps strictly followed the instructions of the kit. The absorbance of A260/A280 is measured by ultra-micro spectrophotometer to ensure that the purity of total RNA is between 1.8 and 2.1 to avoid any pollution.

By utilizing the polyA tail characteristics of eukaryotic mRNA, mRNA was enriched using Oligo (dT) magnetic beads. After treatment with fragmentation buffer, one strand cDNA was synthesized by reverse transcription using random hexamers. During the second strand synthesis, dUTP was used instead of dTTP to construct a chain specific library. Subsequently, end repair, A-tail addition, and adapter connection were performed, followed by magnetic bead purification and screening of a library containing 250–350 bp insertion fragments. After Qubit quantification, Qsep400 fragment analysis, and Q-PCR calibration, 150 bp double ended sequencing was performed on the Illumina platform.

The sequencing data was subjected to fastp quality control to remove reads containing adapters, N ratios > 10%, or low-quality bases (Q ≤ 20) > 50%. Using HISAT to align clean reads to the reference genome and StringTie for predicting new genes. Calculate the logarithmic ratio of genes through featureCounts and convert it into FPKM values to quantify expression levels. Differential analysis was performed using DESeq2, and after Benjamini&Hochberg correction, differentially expressed genes were screened using the corrected P-value and log_2_ fold change threshold. KEGG and GO enrichment analysis were performed based on hypergeometric tests [74].

### RT-qPCR analysis

To verify the accuracy of RNA-seq sequencing, seven differentially expressed genes speculated to be related to thermal exercise to improve the heat tolerance of alfalfa were randomly selected for RT-qPCR verification. The seven differentially expressed genes are *PAL* (Phenylalanine Ammonia-Lyase), *F5H* (Fertilize 5-hydroxylase), *4CL* (4-coumarate Coenzyme A Ligase), *Hsf2b* (Heat stress transcription factor b-2b), *CCR* (Cinnamoyl-coa reduction), *GST* (Glutathione-s-transfer) and Peroxidase. Using β-Actin as the reference gene, the gene-specific primers were designed by PrimerPremer5.0, and the primer sequence is shown in Table S5. RT-qPCR was performed on ABI/Thermo Fisher. There were three independent plants in each of the three biological replicates for all the above experiments. The relative expression of the target gene was measured by 2^−ΔΔCt^ [75].

### Metabolite content detection

Lignin content in leaves and stems of alfalfa was determined by lignin content detection kit (Shanghai Liquid Quality Detection Technology Co., Ltd.). Assay of AsA-GSH activity using commercial kits (Shanghai Liquid Quality Testing Technology Co., Ltd.), the contents of reduced glutathione and ascorbic acid were determined, and the activities of APX, glutathione reductase (GR), monodehydroascorbic acid reductase (MDHAR), dehydroascorbic acid reductase (DHAR) and other enzymes were determined.

### Detection of free fatty acids by proteomics

Samples were thawed on ice, and 50 mg of each sample was transferred to an EP tube. Then, 700 µL of extract was added, and the mixture was vortexed at 2500 rpm for 10 min. Subsequently, ultrasonic treatment was performed at 4 °C for 15 min, followed by centrifugation at 4200 rpm for 5 min. A total of 500 µL of the supernatant was transferred to a glass vial containing 200 µL of 0.05 M sodium hydroxide-methanol solution, and the mixture was dried by nitrogen blowing. The residue was redissolved in 400 µL of 0.4 M sodium hydroxide-methanol solution and incubated at 70 °C for 10 min. After cooling, 500 µL of dichloromethane and 200 µL of double-distilled water were added, and the mixture was vortexed at 2500 rpm for 5 min, then centrifuged at 4200 rpm for 5 min. Next, 300 µL of the supernatant was transferred to a new vial containing 300 µL of 2 M methanolic hydrochloric acid solution, and the mixture was reacted at 70 °C for 20 min for methyl esterification. After cooling, 500 µL of n-hexane and 300 µL of double-distilled water were added, and the mixture was vortexed at 2500 rpm for 5 min, followed by centrifugation at 4200 rpm for 5 min. Finally, the supernatant containing fatty acid methyl esters was collected into an injection vial for GC-MS analysis [76–79].

R language prcomp function, the data is standardized by unit variance and unsupervised principal component analysis is carried out. Taking |Log2FC| as the threshold, the metabolites were annotated in combination with KEGG compound database, mapped to KEGG pathway, and then enriched and analyzed by MSEA.

### Data statistics

Analysis of variance (ANOVA) was performed using IBM SPSS Statistics 22. Plots were made using Origin 2018.

## Supplementary Information

Additional file 1. Table S1 Comprehensive evaluation results of heat tolerance of 12 alfalfa varieties.

Additional file 2. Table S2 Transcriptome sequencing data.

Additional file 3. Table S3 Fatty acid content test results.

Additional file 4. Table S4 Name and source of alfalfa varieties.

Additional file 5. Table S5 Primer sequences.

Supplementary Material 6.

## Data Availability

Raw data was deposited in NCBI database under SRA accession: PRJNA1294583(https://submit.ncbi.nlm.nih.gov/subs/sra/SUB15477709/overview).
