# Supplementary figures and images for: Thermal priming enhances heat tolerance in alfalfa (Medicago sativa L.) through activation of multiple metabolic pathways
Source: BMC Plant Biol. 2025 Dec 9;25:1689. doi: 10.1186/s12870-025-07726-w (PMC12690804; doi:10.1186/s12870-025-07726-w)

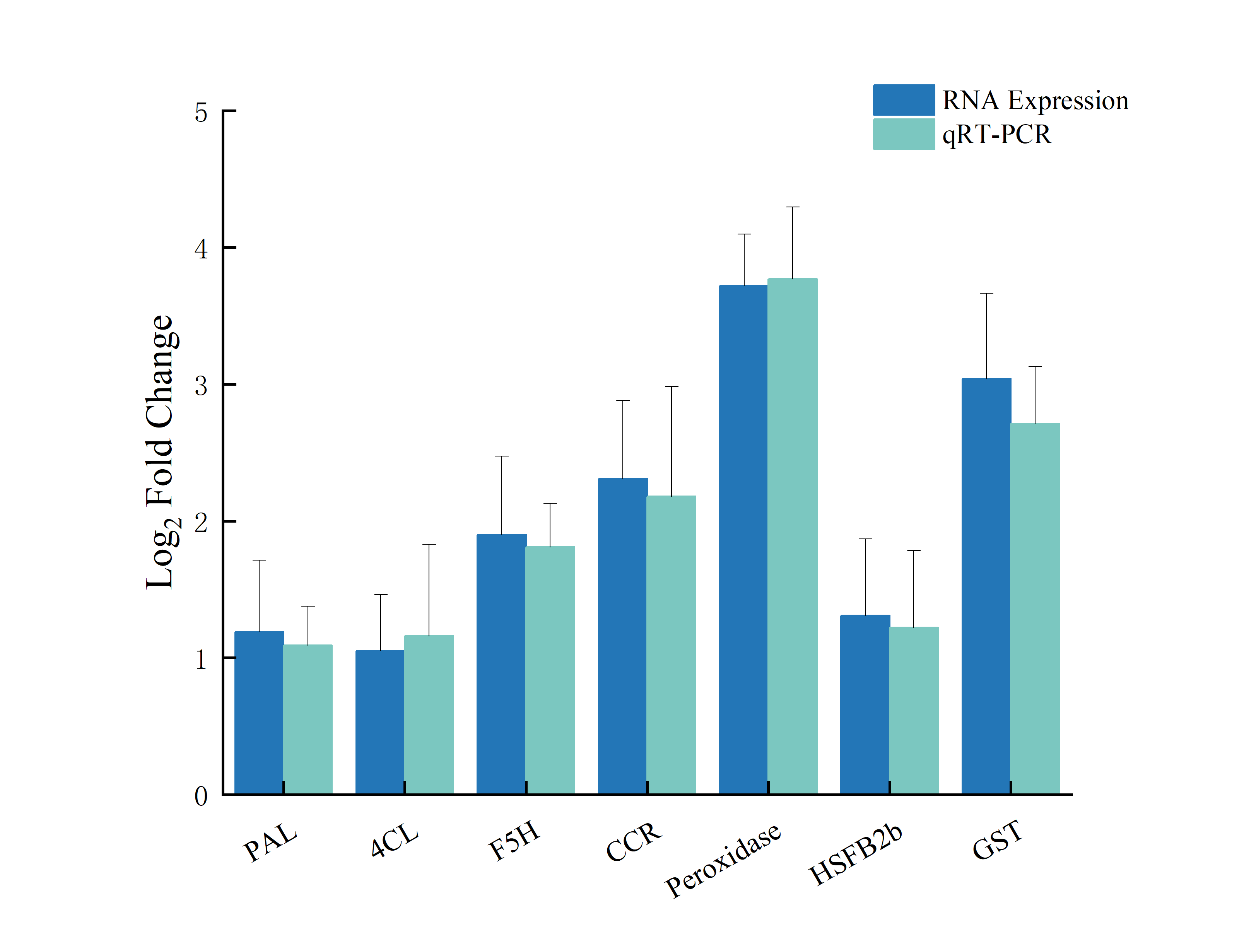


Fig.S1 Validation of gene expression by RT-qPCR analysis.

Supplement: Supplementary file 6 — Supplementary Material 6. [file 12870_2025_7726_MOESM6_ESM.docx]
